# Orientation-Dependent Interfacial Stability and Elastic Anisotropy of α-Fe/Fe_3_C: A First-Principles Study

**DOI:** 10.3390/ma19194147

**Published:** 2026-09-29

**Authors:** Ning Dang, Jianjun Wang, Jia Wang, Yiwen Xu, Junfeng Cao, Hao Tang, Lihong Han

**Affiliations:** 1 State Key Laboratory of Oil and Gas Equipment, CNPC Tubular Goods Research Institute, Xi’an 710077, China; 2 School of Aeronautics, Northwestern Polytechnical University, Xi’an 710072, China; 3 Oil Production Technology Research Institute, PetroChina Xinjiang Oilfield Company, Karamay 831399, China; 4 PetroChina Changqing Oilfield Company, Qingyang 745000, China; 5 School of Geosciences, China University of Petroleum (East China), Qingdao 266580, China

**Keywords:** Fe_3_C, α-Fe/Fe_3_C interface, first-principles calculations, elastic anisotropy, interface energy, work of adhesion

## Abstract

Cementite (Fe_3_C) plays a key role in determining the mechanical performance of pearlitic and heat-resistant steels, where both its intrinsic elastic properties and interfacial bonding with ferrite are critical. In this study, first-principles calculations based on density functional theory were employed to systematically investigate the elastic anisotropy of orthorhombic Fe_3_C and the interfacial stability of α-Fe/Fe_3_C with three typical orientation relationships (Isaichev, Bagaryatsky, and Pitsch–Petch). The calculated elastic constants of Fe_3_C satisfy the mechanical stability criteria and reveal pronounced anisotropic behavior, indicating strong directional dependence of its deformation resistance. Interfacial models with optimized lattice matching demonstrate that the Isaichev orientation exhibits the lowest lattice mismatch (~1.71%), the smallest interface energy (0.55 J/m^2^), and the highest work of adhesion (4.29 J/m^2^), suggesting superior thermodynamic stability and interfacial bonding strength. In contrast, the Bagaryatsky and Pitsch–Petch interfaces exhibit larger lattice mismatch and more pronounced local interfacial distortion, resulting in higher interface energies and reduced interfacial stability. These findings provide atomic-scale insights into the structure–property relationship of Fe_3_C and highlight the critical role of orientation relationships in governing interface stability. The results offer theoretical guidance for interface engineering and microstructure optimization in high-performance steels.

## 1. Introduction

Heat-resistant steels are widely used in power generation, petrochemical equipment, and other high-temperature structural applications, where long-term service performance requires excellent thermal stability, creep resistance, and oxidation resistance [1,2,3,4,5]. In ferritic–pearlitic steels, cementite (Fe_3_C) acts as a key strengthening phase, and its intrinsic mechanical properties, together with its interaction with the ferritic matrix, play a decisive role in determining the overall mechanical performance and service reliability [6].

As a complex orthorhombic intermetallic compound, Fe_3_C exhibits high hardness, strong atomic bonding, and significant lattice distortion, which lead to pronounced anisotropic mechanical behavior [7]. These intrinsic characteristics not only govern its deformation resistance but also influence stress transfer and damage evolution in multiphase steel systems. Therefore, a quantitative understanding of the elastic properties and anisotropy of Fe_3_C is essential for elucidating its strengthening mechanisms and guiding microstructure optimization in advanced steels.

At the microscale, the α-Fe/Fe_3_C interface plays a critical role in load transfer, crack initiation, and resistance to deformation across multiple length scales. Its thermodynamic stability and bonding strength are strongly dependent on the crystallographic orientation relationship between the two phases [8]. Previous studies have investigated the electronic structure and magnetic behavior of Fe_3_C, as well as the orientation relationships in Fe–C systems [9]. Among the orientation relationships reported in ferrite/cementite systems, the Isaichev, Bagaryatsky, and Pitsch–Petch relationships are the most representative coherent or semi-coherent interface configurations experimentally observed in pearlitic and heat-treated steels [10].

The Isaichev orientation relationship is generally characterized by relatively low lattice mismatch and favorable atomic registry, which contributes to enhanced interfacial stability. The Bagaryatsky orientation relationship has been widely reported in ferritic–pearlitic steels and is closely associated with transformation-induced strain accommodation. In contrast, the Pitsch–Petch orientation relationship involves a slight crystallographic inclination between ferrite and cementite lattices, resulting in more complex atomic arrangements and interfacial strain distributions [11,12].

Therefore, these three orientation relationships provide representative atomic-scale models for systematically investigating the influence of crystallographic matching on interfacial bonding behavior, lattice distortion, and thermodynamic stability in α-Fe/Fe_3_C systems [13]. However, these studies mainly focus on either bulk properties or individual interface configurations, and a systematic comparison of interfacial mechanical and energetic characteristics under different orientation relationships remains limited. This gap restricts a comprehensive understanding of the atomic-scale mechanisms governing interfacial stability and mechanical performance in Fe–C systems [14,15].

With the development of density functional theory (DFT), first-principles methods have become powerful tools for investigating the elastic behavior of complex phases and the bonding characteristics of metallic interfaces without reliance on empirical parameters [16]. These methods enable accurate predictions of elastic constants, anisotropic mechanical responses, and interfacial energetics, providing fundamental insights into structure–property relationships at the atomic scale [17,18,19].

In this work, first-principles calculations are employed to systematically investigate the intrinsic elastic properties of orthorhombic Fe_3_C and the interfacial behavior of α-Fe/Fe_3_C under three representative orientation relationships—Isaichev, Bagaryatsky, and Pitsch–Petch. By quantitatively comparing lattice mismatch, interface energy, and work of adhesion, this study aims to elucidate the orientation-dependent mechanisms governing interfacial stability and bonding strength. The results provide atomic-scale insights and offer theoretical guidance for interface engineering and microstructure optimization in high-performance steels [20,21,22,23,24].

## 2. Computational Methods

### 2.1. Density Functional Theory Calculations

First-principles calculations were performed within the framework of density functional theory (DFT) using the Vienna ab initio Simulation Package (VASP). The projector augmented-wave (PAW) method was employed to describe the electron–ion interactions, while the exchange–correlation effects were treated using the generalized gradient approximation (GGA) in the form of the Perdew–Burke–Ernzerhof (PBE) functional. Spin polarization was considered throughout all calculations due to the ferromagnetic nature of α-Fe [25,26,27].

A plane-wave basis set was adopted, with the energy cutoff determined through convergence testing to ensure the accuracy of total energy and stress calculations. A plane-wave cutoff energy of 500 eV was employed after convergence testing to ensure reliable total energies and stress accuracy. The Brillouin zone was sampled using Γ-centered Monkhorst–Pack k-point meshes with a typical density equivalent to 11 × 11 × 11 for bulk calculations, while reduced meshes were adopted for larger interface supercells according to their dimensions. Methfessel–Paxton smearing with a width of 0.1 eV was used during ionic relaxation. Structural optimization was considered converged when the residual Hellmann–Feynman force on each atom was smaller than 0.02 eV/Å.

For interface calculations, convergence tests with respect to slab thickness and vacuum spacing were performed to ensure stable interface energies and eliminate artificial interactions between periodic slabs. Brillouin-zone integrations were carried out using Monkhorst–Pack k-point meshes, which were selected according to the size and symmetry of the computational models. Electronic self-consistency was achieved with an energy convergence criterion of 1.0 × 10^−5^ eV. For large interface supercells, reduced Γ-centered k-point meshes such as 3 × 3 × 1 were employed according to the slab dimensions to balance computational efficiency and accuracy.

### 2.2. Structural Optimization and Elastic Properties

#### 2.2.1. Bulk α-Fe and Fe_3_C

The initial crystal structures of body-centered cubic α-Fe and orthorhombic Fe_3_C (space group Pnma) were obtained from the Materials Project database. All structures were fully relaxed prior to property calculations. Structural optimization was performed until the residual atomic forces were less than 0.02 eV/Å and the total energy change between successive ionic steps was below 1.0 × 10^−5^ eV. The optimized lattice parameters and atomic configurations were subsequently used as the reference states for evaluating mechanical properties [28,29].

#### 2.2.2. Evaluation of Elastic Constants

The elastic properties of Fe_3_C were determined using the stress–strain method based on linear elasticity theory. A series of small finite deformations were applied to the optimized unit cell, and the resulting stress responses were used to extract the full elastic tensor corresponding to its orthorhombic symmetry, which consists of nine independent elastic constants.

Based on the calculated elastic constants, the bulk modulus (B), shear modulus (G), and Young’s modulus (E) were derived using the Voigt–Reuss–Hill averaging scheme. In addition, the elastic anisotropy of Fe_3_C was analyzed using the three-dimensional directional dependence of elastic moduli generated by VASPKIT, providing a comprehensive characterization of its mechanical behavior.

### 2.3. Construction of α-Fe/Fe_3_C Interfaces

#### 2.3.1. Orientation Relationships and Supercell Matching

Three representative orientation relationships commonly reported in Fe–C systems were considered in this study, namely the Isaichev, Bagaryatsky, and Pitsch–Petch relationships. For each case, surface slabs were constructed by cleaving the relaxed bulk structures of α-Fe and Fe_3_C along the corresponding crystallographic planes [30,31,32,33].

To ensure physically meaningful interface configurations, lattice matching between the two phases was achieved using a supercell construction approach. The in-plane lattice parameters of α-Fe and Fe_3_C were adjusted by appropriately expanding their unit cells, with the aim of minimizing lattice mismatch while preserving the crystallographic orientation relationships. This strategy allows for a reasonable balance between structural accuracy and computational efficiency.

A vacuum layer with a thickness of at least 15 Å was introduced along the direction normal to the interface in order to eliminate artificial electrostatic and atomic interactions between periodically repeated slabs under periodic boundary conditions. The constructed interface models therefore consist of two free surfaces and one coherent interface, capturing the essential interfacial bonding characteristics. The lattice mismatch for each interface configuration was quantitatively evaluated to ensure its validity.

#### 2.3.2. Structural Relaxation of Interface Models

The constructed interface supercells were fully relaxed using a two-step optimization procedure. An initial relaxation with relatively loose convergence criteria was first performed to obtain a stable intermediate configuration, followed by a more stringent relaxation to reach the final equilibrium structure.

During the relaxation process, the in-plane lattice vectors were fixed to maintain the predefined orientation relationships, while atomic positions along the direction normal to the interface were fully relaxed. This approach ensures that the interface structure remains crystallographically consistent while allowing sufficient atomic rearrangement to minimize the total energy.

Such a relaxation protocol guarantees that the obtained interface configurations are both energetically stable and mechanically compatible, providing a reliable basis for subsequent calculations of interfacial properties [34,35,36].

### 2.4. Interface Energy and Adhesion Work

The thermodynamic stability and bonding strength of the α-Fe/Fe_3_C interfaces were quantified by calculating the interface energy (γ_int_) and the work of adhesion (W_ad_) [37]. These quantities were obtained from the total energies of the relaxed interface model and the corresponding isolated surface slabs using the following expressions:

(1) $$\gamma_{\mathrm{int}}=\frac{E_{\mathrm{interface}}-E_{\mathrm{slab}}^{\alpha \text{-}\mathrm{Fe}}-E_{\mathrm{slab}}^{{\mathrm{Fe}}_{3}C}}{A}$$

(2) $$W_{\mathrm{ad}}=\frac{E_{\mathrm{slab}}^{\alpha \text{-}\mathrm{Fe}}+E_{\mathrm{slab}}^{{\mathrm{Fe}}_{3}C}-E_{\mathrm{interface}}}{A}$$

where *A* denotes the interfacial area. All energetic quantities were taken from fully relaxed configurations, ensuring a consistent and reliable comparison across the three orientation relationships.

## 3. Structural Optimization and Fundamental Mechanical Properties of Fe and Fe_3_C

The optimized crystal structures and intrinsic mechanical properties of α-Fe and orthorhombic Fe_3_C were systematically investigated based on first-principles calculations. The initial structures were obtained from the Materials Project database and fully relaxed using the PAW–PBE scheme implemented in VASP with Monkhorst–Pack k-point sampling and spin polarization.

The optimized lattice parameters show good agreement with previously reported data, confirming the reliability of the computational approach. For Fe_3_C, the nine independent elastic constants were calculated using the stress–strain method [38]. Based on these results, the bulk modulus, shear modulus, Young’s modulus, and Poisson’s ratio were derived using the Voigt–Reuss–Hill approximation.

Furthermore, the anisotropic mechanical behavior of Fe_3_C was analyzed by visualizing the directional dependence of its elastic properties using VASPKIT [39,40,41]. The results reveal pronounced elastic anisotropy, which originates from the asymmetric atomic arrangement and directional bonding characteristics of the orthorhombic structure. This anisotropic behavior provides important insight into the strengthening role of Fe_3_C and lays the foundation for understanding its influence on the interfacial mechanical response in α-Fe/Fe_3_C systems.

### 3.1. Study of Fundamental Mechanical Properties of Fe_3_C

#### 3.1.1. Construction of the Initial Structural Model

The initial crystal structures of α-Fe and Fe_3_C were constructed based on data obtained from the Materials Project database to ensure the reliability of the starting configurations. The α-Fe phase adopts a body-centered cubic (BCC) structure with lattice parameters of a = b = c = 2.83135 Å, which is well established in both experimental and theoretical studies.

Cementite (Fe_3_C), as a key intermediate phase in Fe–C alloys, exhibits a complex crystal structure and plays a crucial role in determining the mechanical properties of steels [42,43,44,45]. Among its possible polymorphs, the orthorhombic phase (space group Pnma) is thermodynamically more stable and commonly observed under typical conditions [10]. Therefore, orthorhombic Fe_3_C was selected for subsequent calculations.

The corresponding lattice parameters and atomic configurations of Fe_3_C were adopted from previous studies [10], with lattice constants of a = 5.092 Å, b = 6.741 Å, and c = 4.527 Å. The constructed unit cell models of α-Fe and Fe_3_C are summarized in Table 1, and their crystal structures are illustrated in Figure 1.

#### 3.1.2. Structural Optimization of the Initial Models

Structural optimization of α-Fe and Fe_3_C was carried out within the framework of density functional theory using the PAW–PBE approach implemented in VASP. Spin polarization was included to account for the magnetic nature of Fe-containing systems [46].

During the relaxation process, the atomic positions and lattice parameters were fully optimized until the convergence criteria were satisfied, ensuring that the obtained structures correspond to stable equilibrium configurations. The optimized lattice parameters for both α-Fe and Fe_3_C are presented in Table 2.

The optimized α-Fe structure retains its body-centered cubic symmetry with lattice parameters consistent with reference values, while Fe_3_C preserves its orthorhombic (Pnma) structure. The calculated lattice constants show good agreement with previously reported theoretical and experimental data [10], confirming the reliability of the computational setup [47,48,49].

Overall, the optimized structures provide a robust basis for subsequent calculations of elastic properties and interfacial behavior.

### 3.2. Calculation of Mechanical Properties of Fe_3_C

Subsequently, the mechanical properties of the optimized Fe_3_C were evaluated using the stress–strain method within the framework of linear elasticity theory [48]. In this approach, a series of small strains were applied to the structure, and the corresponding stress responses were calculated to determine the elastic constants based on Hooke’s law.

The calculations were performed using appropriate computational settings in VASP, and the resulting stress–strain data were extracted and processed using the VASPKIT program [50]. The nine independent elastic constants of Fe_3_C obtained from these calculations are listed in the table below.

As shown in Table 3, Fe_3_C belongs to the orthorhombic crystal system (space group Pnma) and is characterized by nine independent elastic constants: C_11_, C_22_, C_33_, C_44_, C_55_, C_66_, C_12_, C_13_, and C_23_. The calculated results are analyzed according to the mechanical stability criteria for orthorhombic crystals.

$$\left[\begin{array}{cccccc} C_{11} & C_{12} & C_{13} & 0 & 0 & 0\\ C_{12} & C_{22} & C_{23} & 0 & 0 & 0\\ C_{13} & C_{23} & C_{33} & 0 & 0 & 0\\ 0 & 0 & 0 & C_{44} & 0 & 0\\ 0 & 0 & 0 & 0 & C_{55} & 0\\ 0 & 0 & 0 & 0 & 0 & C_{66} \end{array}\right]$$

The diagonal elastic constants are all positive:

C_11_ = 377.5 > 0, C_22_ = 342.3 > 0, C_33_ = 305.2 > 0,

C_44_ = 14.8 > 0, C_55_ = 134.5 > 0, C_66_ = 132.3 > 0.

Furthermore, the following linear combination conditions are satisfied:

C_11_ + C_22_ − 2C_12_ = 377.5 + 342.3 − 2 × 156.3 = 407.2 > 0,

C_11_ + C_33_ − 2C_13_ = 377.5 + 305.2 − 2 × 153.3 = 376.1 > 0,

C_22_ + C_33_ − 2C_23_ = 342.3 + 305.2 − 2 × 170.6 = 306.4 > 0,

C_11_ + C_22_ + C_33_ + 2(C_12_ + C_13_ + C_23_) = 1025.0 + 2(156.3 + 153.3 + 170.6) = 1929.6 > 0.

According to the mechanical stability criteria for orthorhombic crystals [51], all calculated elastic constants satisfy the required inequality conditions, including diagonal positivity, linear combination constraints, and energy positive definiteness. These results indicate that Fe_3_C is mechanically stable under equilibrium conditions. In addition, the calculated values are in good agreement with previously reported data [10], confirming the reliability of the present calculations.

The bulk modulus (B) and shear modulus (G) were calculated from the elastic constants using the Voigt–Reuss–Hill (VRH) approximation [11,52], which provides a reliable estimation of the effective elastic properties of polycrystalline materials.

For the orthorhombic system, the Voigt and Reuss bounds are given as follows:

Voigt model:

(3) $$B_{v}=\frac{C_{11}+2C_{12}}{3}\;G_{v}=\frac{C_{11}-C_{12}+3C_{44}}{5}$$

Reuss model:

(4) $$B_{R}=\frac{C_{11}+2C_{12}}{3}\;G_{R}=\frac{5C_{44}(C_{11}-C_{12})}{3(C_{11}-C_{12})+4C_{44}}$$

The effective bulk modulus (B) and shear modulus (G) were then obtained by averaging the Voigt and Reuss limits according to the Hill scheme:

Hill model:

(5) $$B=\frac{B_{v}+B_{R}}{2}\;G=\frac{G_{v}+G_{R}}{2}$$

Based on the obtained values of B and G, the Young’s modulus (E) and Poisson’s ratio (ν) were further derived using the following relationships:

(6) $$E=\frac{9BG}{3B+G}\;V=\frac{3B-2G}{2(3B+G)}$$

Using the above equations, the macroscopic mechanical properties of Fe_3_C were determined, and the results are summarized in Table 4.

The relatively large difference between the Voigt and Reuss shear moduli indicates pronounced elastic anisotropy in orthorhombic Fe_3_C. The Voigt approximation assumes uniform strain throughout the polycrystalline aggregate, whereas the Reuss approximation assumes uniform stress. For highly anisotropic materials with directional bonding heterogeneity, these two assumptions may lead to significantly different predictions of shear response. Therefore, the large Voigt–Reuss deviation observed here further confirms the strong elastic anisotropy and directional mechanical heterogeneity of Fe_3_C.

Figure 2 presents the three-dimensional spatial distribution of the mechanical response of Fe_3_C, including the bulk modulus, Young’s modulus, shear modulus (maximum and minimum), and Poisson’s ratio. Pronounced elastic anisotropy is clearly observed in all mechanical parameters. Representative parameters including bulk modulus, Young’s modulus, shear modulus, and Poisson’s ratio were selected for visualization because they directly characterize the most important elastic responses associated with compression, tension, shear deformation, and lateral strain behavior, respectively. Although all distributions are derived from the same Fe_3_C crystal structure, different mechanical parameters correspond to distinct tensor components and deformation modes. Bulk modulus reflects resistance to volumetric compression, Young’s modulus describes tensile stiffness, shear modulus characterizes resistance to shear deformation, while Poisson’s ratio represents transverse strain response. Because these mechanical responses are governed by different combinations of elastic tensor components, their directional distributions exhibit different three-dimensional shapes despite originating from the same orthorhombic crystal structure.

As shown in Figure 2a, the bulk modulus exhibits a relatively smooth but direction-dependent distribution, with higher values mainly distributed along the x-axis and several diagonal orientations, whereas relatively lower values appear near the z-direction. This indicates stronger resistance to volumetric compression along the z-direction, which can be attributed to the directional variation in bonding stiffness within the crystal structure [49].

The Young’s modulus, shown in Figure 2b, displays a more pronounced anisotropic distribution, ranging from approximately 55 GPa to 308 GPa. The highest values are observed along the z-axis and several diagonal directions, whereas lower values appear in perpendicular orientations, resulting in a typical petal-like pattern. This significant variation reflects strong directional dependence of tensile stiffness and originates from the anisotropic chemical bonding between non-equivalent atoms in Fe_3_C.

Figure 2c illustrates the distribution of the maximum shear modulus, where the highest values are concentrated along specific crystallographic directions, reaching up to 134 GPa. This suggests that Fe_3_C possesses strong shear resistance on certain crystallographic planes, which may correspond to preferred slip systems during deformation.

In contrast, Figure 2d reveals a distinct cross-shaped region with significantly lower shear modulus, with minimum values of approximately 15 GPa. These low-modulus directions indicate weak shear resistance and are likely to act as preferred pathways for plastic deformation or crack propagation under external loading [53,54,55].

The Poisson’s ratio distribution shown in Figure 2e exhibits a complex three-dimensional pattern, with values ranging from approximately 0.31 to 1.26, indicating strong anisotropy in lateral deformation behavior. The unusually high directional Poisson’s ratio observed in several orientations may originate from the strong elastic anisotropy and directional coupling effects associated with the orthorhombic crystal symmetry of 3C. Similar anomalous directional responses have also been reported in highly anisotropic intermetallic compounds and low-symmetry crystalline systems. Nevertheless, the microscopic origin of this behavior still requires further investigation through more detailed electronic structure and phonon analyses. Directions with higher Poisson’s ratio correspond to significant lateral contraction under uniaxial tension, which may influence dimensional stability and structural integrity.

Overall, the strong anisotropic mechanical behavior of 3C arises from its asymmetric atomic arrangement and directional bonding characteristics. Such anisotropy plays a critical role in governing deformation behavior and is expected to significantly influence stress transfer and mechanical compatibility at α-Fe/3C interfaces.

## 4. Study of the Mechanical Properties of the α-Fe_3_C Interface

The interfacial bonding characteristics between α-Fe and 3C play a crucial role in determining the synergistic mechanical response and thermodynamic stability of Fe–C systems. In particular, the strength and stability of the interface directly influence stress transfer, deformation compatibility, and overall mechanical performance.

In this study, three representative orientation relationships commonly observed in Fe_3_C/α-Fe systems were selected, namely the Isaichev orientation (Fe_3_C (101)/Fe (11$\bar{2}$)), the Bagaryatsky orientation (Fe_3_C (001)/Fe (11$\bar{2}$)), and the Pitsch–Petch orientation (Fe_3_C (001)/Fe ($\bar{2}\bar{1}$5)). Based on these configurations, corresponding surface slab models and interface supercells were constructed.

To ensure physically meaningful interface structures, the lattice mismatch between the two phases was carefully controlled, and a vacuum layer with a thickness of at least 15 Å was introduced along the direction normal to the interface. Structural optimization was performed using a two-step relaxation strategy to achieve full convergence in both energy and atomic forces.

Subsequently, the interfacial properties were quantitatively evaluated by calculating the interface energy and work of adhesion. Through a comparative analysis of different orientation relationships, the thermodynamic stability and bonding strength of the interfaces were systematically assessed. The results provide atomic-scale insights into orientation-dependent interfacial behavior and offer theoretical guidance for interface engineering in Fe–C systems [56,57].

### 4.1. Model Construction

#### 4.1.1. Surface Model Construction

The slab model was employed to simulate the surface and interfacial properties of α-Fe/Fe_3_C systems. This approach constructs a finite number of atomic layers extracted from a periodic crystal, thereby introducing free surfaces while preserving in-plane periodicity, which is essential for accurately capturing surface effects [58].

To ensure reliable computational results, the thickness of the slab and the vacuum layer were carefully optimized. A sufficiently large vacuum region was introduced along the direction normal to the surface to eliminate spurious interactions between periodic images. In this study, a vacuum layer of 15 Å was adopted for all surface models.

Three representative α-Fe/Fe_3_C orientation relationships were considered, namely the Isaichev orientation (Fe_3_C (101)/Fe (11$\bar{2}$)), the Bagaryatsky orientation (Fe_3_C (001)/Fe (11$\bar{2}$)), and the Pitsch–Petch orientation (Fe_3_C (001)/Fe ($\bar{2}\bar{1}$5)) [11]. For each orientation, the corresponding surface slabs were constructed separately prior to interface assembly.

Considering the requirement of lattice matching between the two phases, the supercell method under periodic boundary conditions was employed to construct the surface models. This approach allows for a realistic representation of the crystal environment while minimizing lattice mismatch between α-Fe and Fe_3_C [59]. To avoid artificial interactions along the surface normal direction, a vacuum layer of 15 Å was consistently applied.

In addition, sufficient slab thickness was ensured so that atoms in the central region exhibit bulk-like behavior. Meanwhile, the number of atomic layers was minimized to balance computational efficiency and physical accuracy. The constructed surface models for the three orientation relationships are illustrated in Figure 3.

#### 4.1.2. Interface Model Construction

Interface models were constructed for three representative α-Fe/Fe_3_C orientation relationships, namely the Isaichev orientation (Fe_3_C (101)/Fe (11$\bar{2}$)), the Bagaryatsky orientation (Fe_3_C (001)/Fe (11$\bar{2}$)), and the Pitsch–Petch orientation (Fe_3_C (001)/Fe ($\bar{2}\bar{1}$5)).

For each orientation, the corresponding crystallographic planes and directions of α-Fe and Fe_3_C were first identified based on crystallographic analysis. The interface configurations were then established through lattice matching and appropriate rotational alignment to ensure structural compatibility between the two phases [60]. On this basis, supercell models were constructed under periodic boundary conditions to maintain in-plane continuity and symmetry.

During model construction, particular attention was paid to minimizing lattice mismatch between the two phases. The in-plane lattice parameters were adjusted by varying the repetition of unit cells and applying necessary shear transformations, ensuring that the mismatch ratio was controlled within an acceptable range (typically below 5%). For unavoidable strain, deformation was preferentially assigned to the phase with higher mechanical stiffness to reduce structural distortion and its impact on interfacial energy and property calculations [61].

To eliminate artificial interactions between periodic images along the interface normal direction, a vacuum layer of at least 15 Å was introduced in all interface models. After construction, full atomic relaxation was performed to obtain energetically stable configurations, allowing atoms near the interface to reach equilibrium positions and accurately reflect interfacial bonding characteristics.

The interface model for the Isaichev orientation is illustrated in Figure 4.

For the Bagaryatsky orientation, defined by Fe_3_C (001)/Fe (11$\bar{2}$), the interface model was constructed following the same general procedure. The corresponding crystallographic planes were selected to maintain consistency with the orientation relationship, and a vacuum layer of 15 Å was introduced along the interface normal direction to eliminate interactions between periodic images. The resulting interface structure is shown in Figure 5.

For the Pitsch–Petch orientation (Fe_3_C (001)/Fe ($\bar{2}\bar{1}$5)), the interface construction additionally involves a small inclination angle of approximately 2.6°, which was carefully preserved during model generation to maintain geometric consistency. The interface model was then constructed using the same approach, with appropriate lattice matching and a vacuum layer of 15 Å applied. The final interface configuration is presented in Figure 6.

The completed interface models for the three different orientations are shown in Figure 7.

To achieve effective lattice matching at the α-Fe/Fe_3_C interface, the in-plane lattice parameters of the two phases were appropriately adjusted along the u and v directions. The lattice mismatch was evaluated by comparing the lattice constants of the two materials along the corresponding crystallographic directions. The mismatch degree is defined as follows:

(7) $$\varepsilon =\frac{1}{2}\left(\frac{a_{1}^{film}-a_{1}^{sub}}{a_{1}^{sub}}+\frac{a_{2}^{film}-a_{2}^{sub}}{a_{2}^{sub}}\right)\times 100\%$$

where a^film^ and a^sub^ represent the lattice constants of the film (Fe_3_C) and substrate (α-Fe), respectively, and a_1_ and a_2_ denote the lattice parameters along the two in-plane crystallographic directions. The calculated lattice parameters and corresponding mismatch degrees for the three orientation relationships are summarized in Table 5.

The interfacial mismatch strains for the three typical orientation relationships follow the order: Pitsch–Petch (12.6%) > Bagaryatsky (7.212%) > Isaichev (1.712%). This result indicates a significant variation in lattice compatibility among the different interface configurations.

Among the reported Fe_3_C/α-Fe interface structures, the Isaichev orientation exhibits the lowest mismatch strain, which is in good agreement with previously reported theoretical values [62]. This low mismatch suggests excellent lattice compatibility between the two phases, facilitating the formation of a stable interface with reduced interfacial stress.

In contrast, the Pitsch–Petch orientation shows a substantially higher mismatch, indicating severe lattice distortion at the interface. Such a high mismatch is expected to induce significant interfacial stress during the bonding process, which may not only affect structural stability but also lead to local lattice distortion and deviations in the interface orientation angle. This behavior is consistent with experimental observations. Experimentally, the Isaichev and Bagaryatsky orientation relationships are more frequently observed in pearlitic and tempered steels because their relatively lower mismatch facilitates interface formation during phase transformation and heat treatment. In contrast, the Pitsch–Petch orientation is less commonly reported owing to its larger crystallographic incompatibility and higher interfacial strain [63]. The preferred orientation relationship in practical steels is strongly influenced by alloy composition, cooling rate, transformation kinetics, and heat-treatment conditions.

### 4.2. Structural Optimization of Interface Models

After constructing the surface and interface models, structural optimization was performed to obtain energetically stable configurations and ensure reliable evaluation of interfacial properties. All calculations were carried out using a plane-wave basis set with an energy cutoff of 450 eV [11]. Brillouin-zone sampling was performed using Monkhorst–Pack k-point meshes, with a grid precision of 0.03 Å^−1^ and a single k-point along the z-direction due to the slab geometry. Spin polarization was included to account for the magnetic nature of Fe.

The convergence criteria for the electronic self-consistent calculations and ionic relaxation were set to 1.0 × 10^−5^ eV and 1.0 × 10^−3^ eV/Å, respectively, ensuring that all structures were fully relaxed to stable equilibrium states.

To improve computational efficiency for large interface systems, a two-step relaxation strategy was adopted. An initial coarse optimization was performed to obtain a reasonable configuration, followed by a refined optimization to achieve higher accuracy. This approach effectively balances computational cost and result reliability [64].

### 4.3. Calculation of Interface Strength and Stability

To quantitatively evaluate the thermodynamic stability and bonding strength of the α-Fe/Fe_3_C interfaces, the interface energy (γ_int_) and the work of adhesion (W_ad_) were calculated based on the fully relaxed interface models.

#### Interface Energy and Work of Separation

To ensure that atoms in the inner region of the slab model exhibit bulk-like behavior, a sufficient number of atomic layers must be included. However, increasing the slab thickness significantly raises computational cost. Therefore, convergence tests were performed to determine an appropriate layer thickness, achieving a balance between computational efficiency and structural reliability.

Surface energy is an important physical quantity used to evaluate the stability of a material surface, defined as the energy required to create a new surface per unit area. By calculating the surface energy for different slab thicknesses and analyzing its convergence behavior, the optimal number of layers for the surface model can be determined.

In interface studies, the work of separation (W_sep_) is widely used to characterize interfacial bonding strength and adhesion properties. It is defined as the reversible work per unit area required to separate a bonded interface into two independent free surfaces. A higher value of W_sep_ indicates stronger interfacial bonding and greater thermodynamic stability. The work of separation is calculated as follows:

(8) $$W_{sep}=\frac{E_{\alpha }+E_{\beta }-E_{\alpha /\beta }}{A}$$

where E_α_ and E_β_ are the total energies of the two corresponding free surfaces after full relaxation, E_α/β_ represents the total energy of the optimized interface system, and A is the interfacial area.

In addition to the work of separation, the interface energy E_interface_ is another key parameter for evaluating the thermodynamic stability of an interface. It represents the excess energy per unit area associated with interface formation, primarily arising from changes in atomic coordination and lattice distortion at the interface.

For homogeneous systems or materials with similar lattice parameters, the interface energy is typically negligible. In contrast, for heterogeneous systems composed of structurally different phases, the interface energy is generally positive due to the presence of lattice mismatch and interfacial strain.

If the interface energy becomes negative, the interface is thermodynamically unstable, indicating a tendency for the system to evolve toward a lower-energy configuration. Such instability may promote atomic diffusion across the interface and even trigger interfacial reactions, potentially leading to the formation of new phases.

The interface energy is calculated using the following expression [11]:

(9) $$\begin{array}{l} E_{\mathrm{interface}}=\frac{1}{A}\left(E_{total}-E_{phase1}-E_{phase2}\right)-\gamma_{phase1}-\gamma_{phase2}\\ =\frac{1}{A}\left(E_{total}-\sum^{i}N_{i}\mu_{i}-\sum^{j}N_{j}\mu_{j}\right)-\frac{1}{2A}\left(E_{phase1}-\sum^{i}N_{i}\mu_{i}\right)-\frac{1}{2A}\left(E_{phase2}-\sum^{j}N_{j}\mu_{j}\right) \end{array}$$

where *E*_total_ represents the total energy of the interface system, and *E*_phase1_ and *E*_phase2_ denote the total energies of the two constituent phases, respectively. *γ* is the surface energy, and *A* is the interfacial area. To eliminate the contribution of surface energy, terms involving chemical potentials are introduced in the expansion, where *N_i_* represents the number of atoms of element *I*, and *μ_i_* corresponds to the chemical potential of the bulk phase.

Based on the above formulation, the interface energies for different orientation relationships can be determined. Combined with the calculation of the work of separation, the interfacial energy and adhesion properties of the three interface configurations were obtained, as summarized in Table 6.

For a clearer comparison, the interface energies and works of separation of the three representative orientation relationships are presented in Figure 8.

It should be noted that the absolute total energies (Etotal) of different interface systems cannot be directly compared because the corresponding supercells contain different numbers of atoms, slab thicknesses, and interface areas. In particular, the Pitsch–Petch interface model contains a substantially larger supercell, resulting in a much lower absolute total energy. Therefore, normalized quantities such as interface energy and work of separation are more physically meaningful descriptors for evaluating interfacial stability and bonding characteristics. A comparative analysis of Table 6 reveals that the interface energy follows the order: Isaichev (0.55 J/m^2^) < Bagaryatsky (1.24 J/m^2^) < Pitsch–Petch (1.46 J/m^2^), indicating significant differences in thermodynamic stability among the three interface configurations. The Isaichev interface exhibits the lowest interface energy, suggesting that its formation is energetically the most favorable and associated with higher structural stability. In contrast, the Pitsch–Petch interface shows the highest interface energy, indicating a less favorable formation process and reduced thermodynamic stability.

The work of separation follows the order: Isaichev (4.29 J/m^2^) > Pitsch–Petch (3.72 J/m^2^) > Bagaryatsky (3.47 J/m^2^). The higher work of separation for the Isaichev interface indicates stronger interfacial bonding and greater resistance to fracture, reflecting a more stable and cohesive atomic configuration. The present results are generally consistent with previously reported theoretical and experimental trends for ferrite/cementite interfaces, particularly regarding the superior stability of the Isaichev orientation relationship [65]. Previous studies have similarly indicated that interfaces with lower lattice mismatch tend to exhibit lower interface energy and stronger adhesion behavior. Minor quantitative deviations may originate from differences in slab thickness, interface termination, exchange–correlation functionals, and lattice matching schemes employed in different calculations. Conversely, the relatively lower work of separation for the Bagaryatsky interface suggests weaker interfacial bonding, which may be attributed to reduced atomic coordination and mismatch-induced local lattice distortion near the interface region.

Although the three interface configurations exhibit generally similar atomic stacking characteristics, their interfacial energetics differ significantly because the local atomic registry, lattice mismatch, and strain accommodation mechanisms are different for each orientation relationship. In the Isaichev configuration, the relatively small lattice mismatch enables better crystallographic matching between α-Fe and Fe_3_C, thereby reducing interfacial strain accumulation and promoting stronger Fe–Fe and Fe–C bonding across the interface. Consequently, the Isaichev interface exhibits the lowest interface energy and the highest work of separation.

By contrast, the Bagaryatsky and Pitsch–Petch interfaces contain larger mismatch-induced distortions and less favorable atomic coincidence at the interface plane. These structural incompatibilities generate localized stress concentration and weaken interfacial bonding strength, resulting in increased interface energy and reduced adhesion behavior. Particularly for the Pitsch–Petch orientation, the large mismatch and crystallographic inclination further aggravate geometric incompatibility, leading to the highest interface energy among the three configurations. In contrast, although the Pitsch–Petch interface shows relatively high bonding strength, its large interface energy suggests that its overall stability is limited. The Bagaryatsky interface exhibits intermediate behavior without a clear advantage in either parameter.

Overall, these results indicate that the interfacial properties of the α-Fe/Fe_3_C system are strongly influenced by the combined effects of lattice mismatch, atomic bonding configuration, and interfacial strain. From an interface engineering perspective, the Isaichev orientation is therefore the most favorable configuration for achieving improved interfacial stability and bonding performance.

## 5. Conclusions

In this study, first-principles calculations were performed to investigate the intrinsic mechanical properties of cementite (Fe_3_C) and the interfacial characteristics of α-Fe/Fe_3_C systems under three representative orientation relationships. The optimized structures of α-Fe and Fe_3_C are in good agreement with reported data, confirming the reliability of the computational approach. The calculated elastic constants of Fe_3_C satisfy the mechanical stability criteria for orthorhombic crystals and reveal pronounced elastic anisotropy, which is closely related to its non-cubic crystal symmetry.

Three interface models—Isaichev, Bagaryatsky, and Pitsch–Petch—were constructed and fully relaxed. The results demonstrate a clear dependence of interfacial properties on crystallographic orientation. Among the three configurations, the Isaichev interface exhibits the lowest lattice mismatch, the lowest interface energy, and the highest work of adhesion, indicating superior thermodynamic stability and the strongest interfacial bonding. In contrast, the Bagaryatsky and Pitsch–Petch interfaces show higher mismatch and increased structural distortion, leading to reduced stability and weaker interfacial bonding.

The present findings provide theoretical guidance for controlling ferrite/cementite interface stability through crystallographic orientation engineering. These results may contribute to the design of high-strength pearlitic steels, heat-resistant steels, and interface-dominated multiphase alloys with enhanced mechanical compatibility and improved resistance to interfacial failure.

Overall, the interfacial behavior of the α-Fe/Fe_3_C system is governed by the combined effects of lattice mismatch, atomic bonding configuration, and interfacial strain. These findings establish a clear structure–property relationship and provide theoretical guidance for optimizing interface design and improving microstructural stability in Fe–C-based materials. The present findings may provide useful theoretical support for designing high-strength pearlitic steels, wear-resistant steels, and heat-resistant structural alloys with improved interface stability and mechanical compatibility.

## Figures and Tables

**Figure 1:**
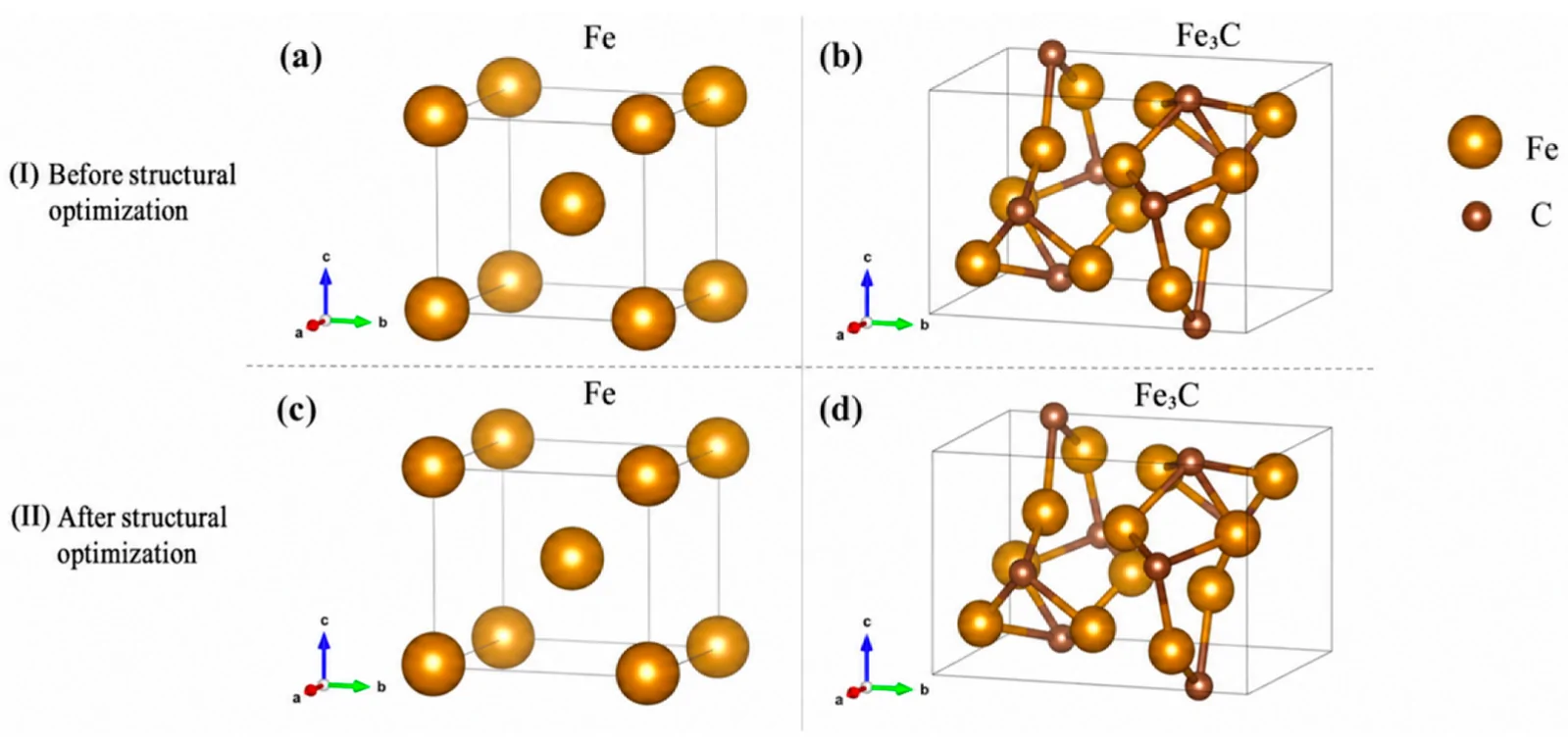
Unit cell structures of Fe and Fe_3_C before and after structural optimization: (**I**) before optimization; (**II**) after optimization. (**a**,**b**) Fe and Fe_3_C before optimization; (**c**,**d**) Fe and Fe_3_C after optimization.

**Figure 2:**
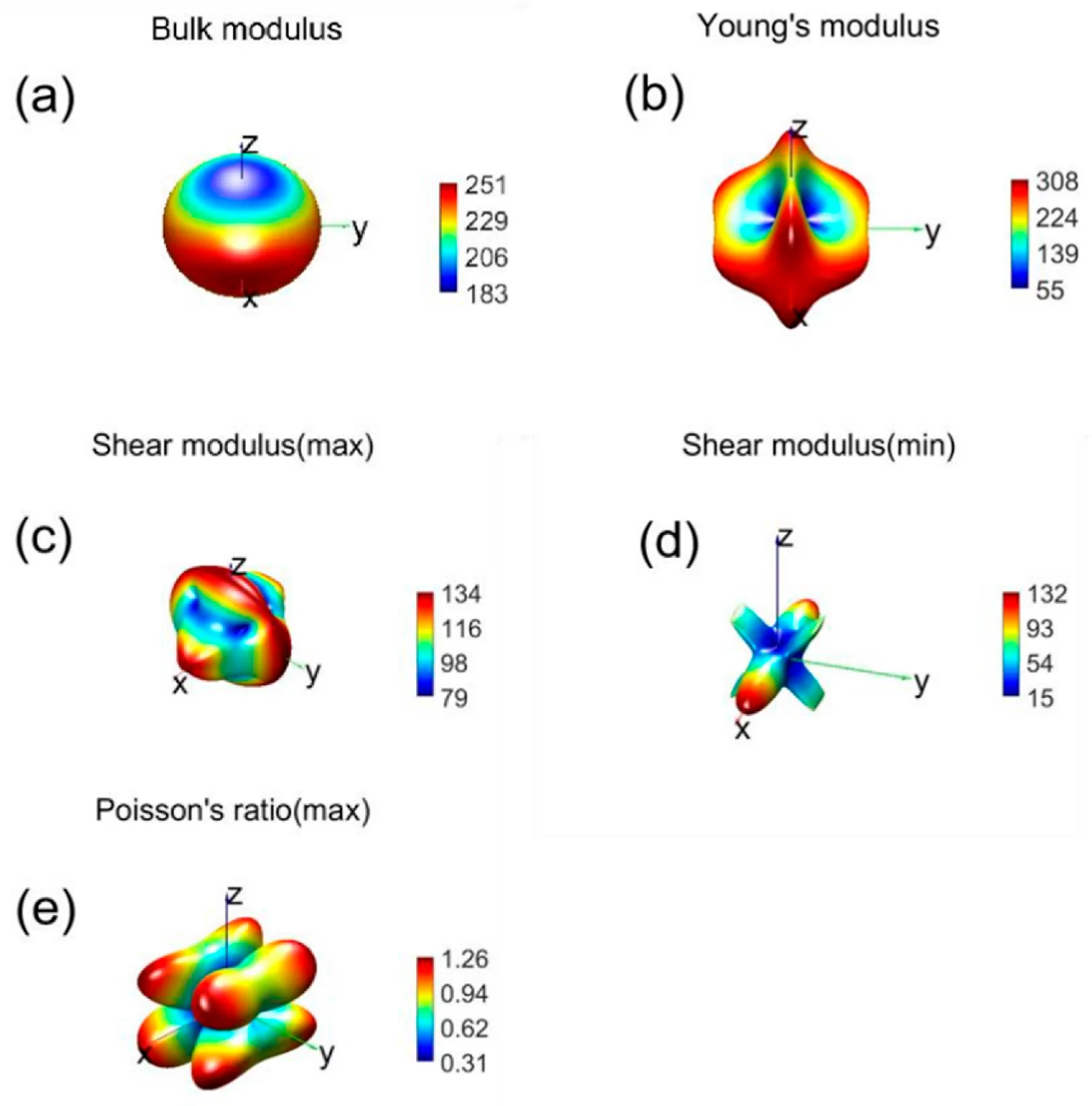
Three-dimensional visualization of the anisotropic distribution of mechanical parameters for Fe_3_C.

**Figure 3:**
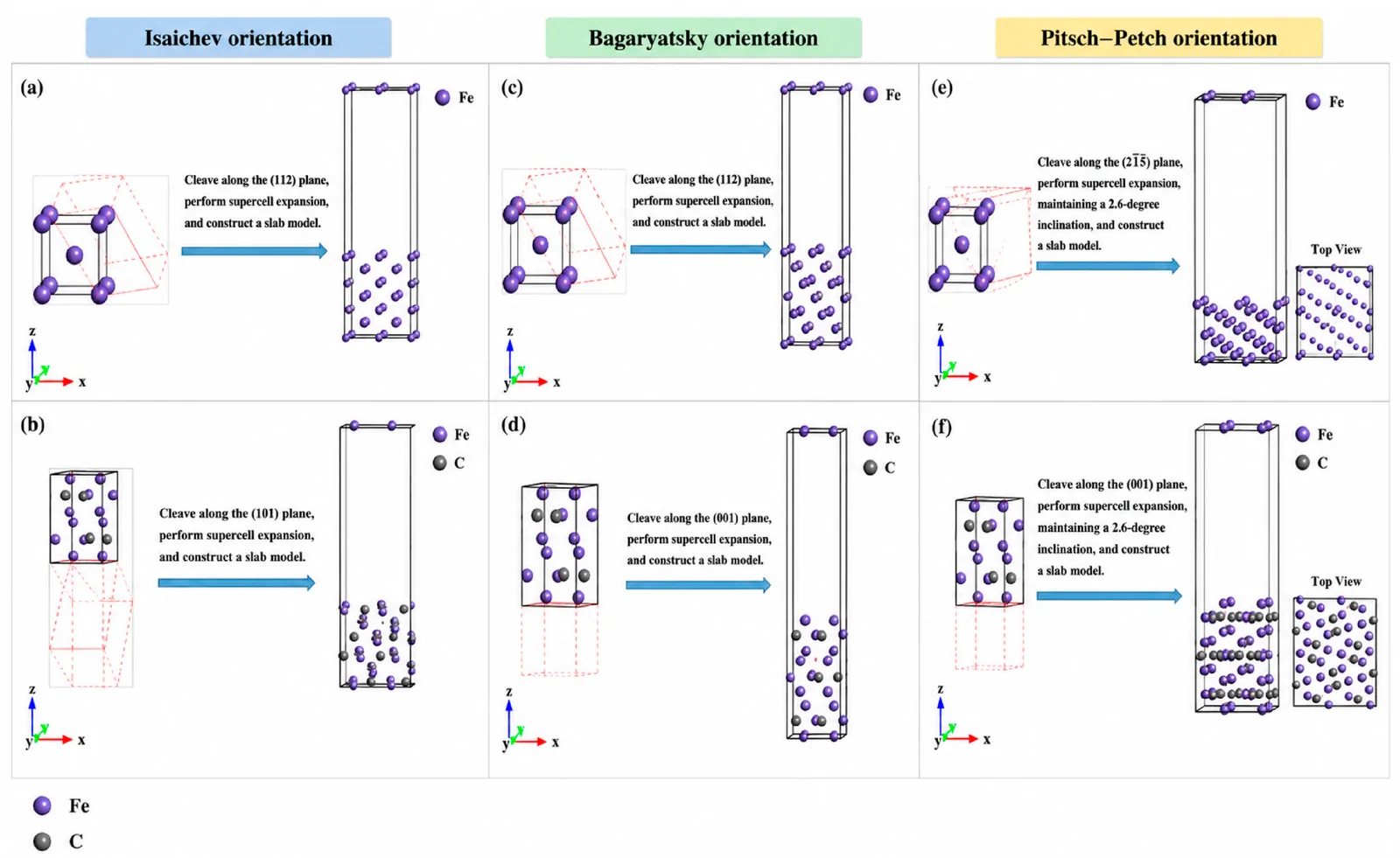
Surface slab models for the three representative α-Fe/Fe_3_C orientation relationships. (**a**) α-Fe slab cleaved along the (11$\bar{2}$)) plane for the Isaichev orientation; (**b**) Fe_3_C slab cleaved along the (101) plane for the Isaichev orientation; (**c**) α-Fe slab cleaved along the (11$\bar{2}$) plane for the Bagaryatsky orientation; (**d**) Fe_3_C slab cleaved along the (001) plane for the Bagaryatsky orientation; (**e**) α-Fe slab cleaved along the ($\bar{2}$)$\bar{1}$5) plane with a 2.6° inclination for the Pitsch–Petch orientation; (**f**) Fe_3_C slab cleaved along the (001) plane with a 2.6° inclination for the Pitsch–Petch orientation.

**Figure 4:**
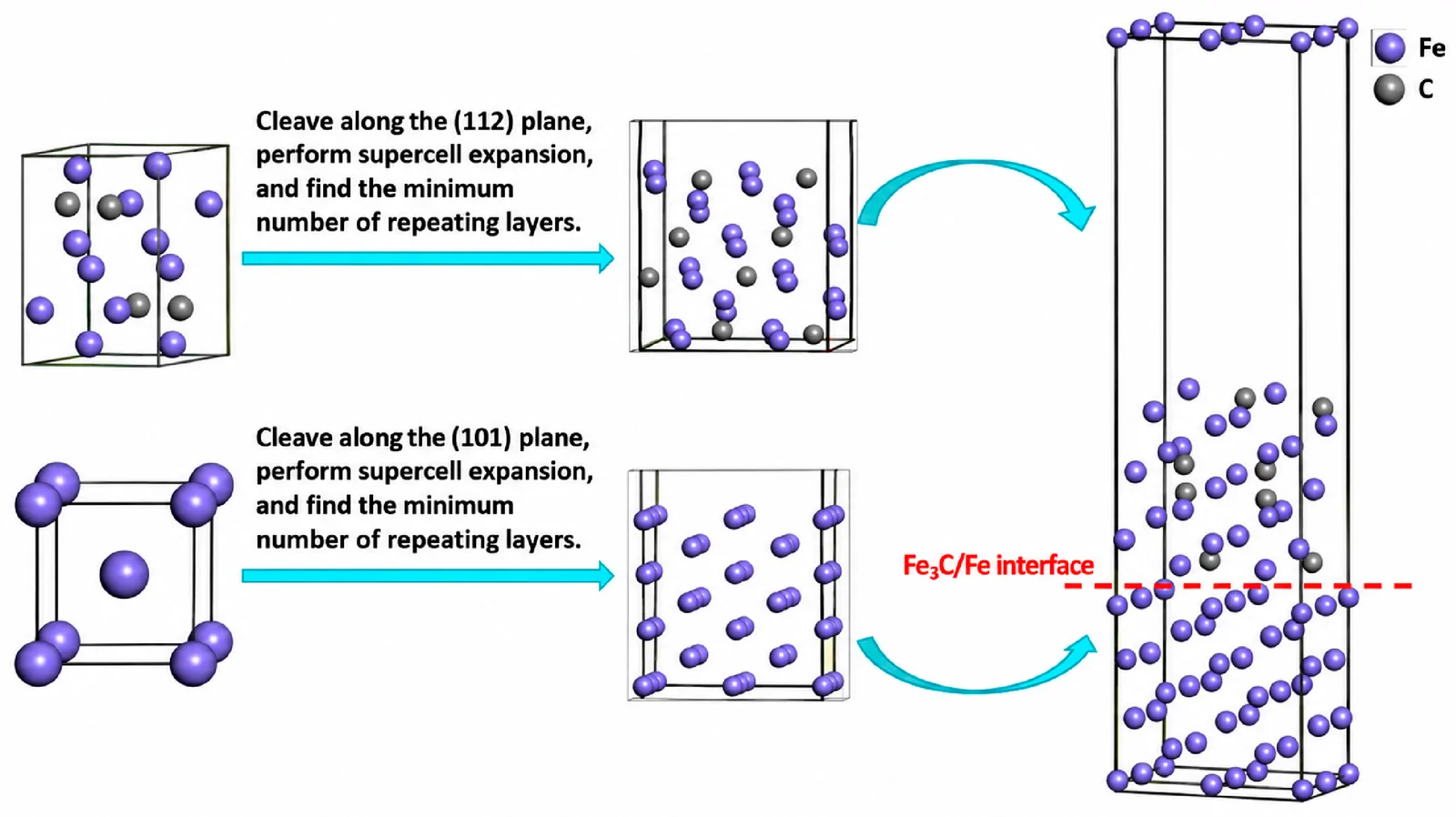
Construction process of the Fe and Fe_3_C interface model in the Isaichev orientation.

**Figure 5:**
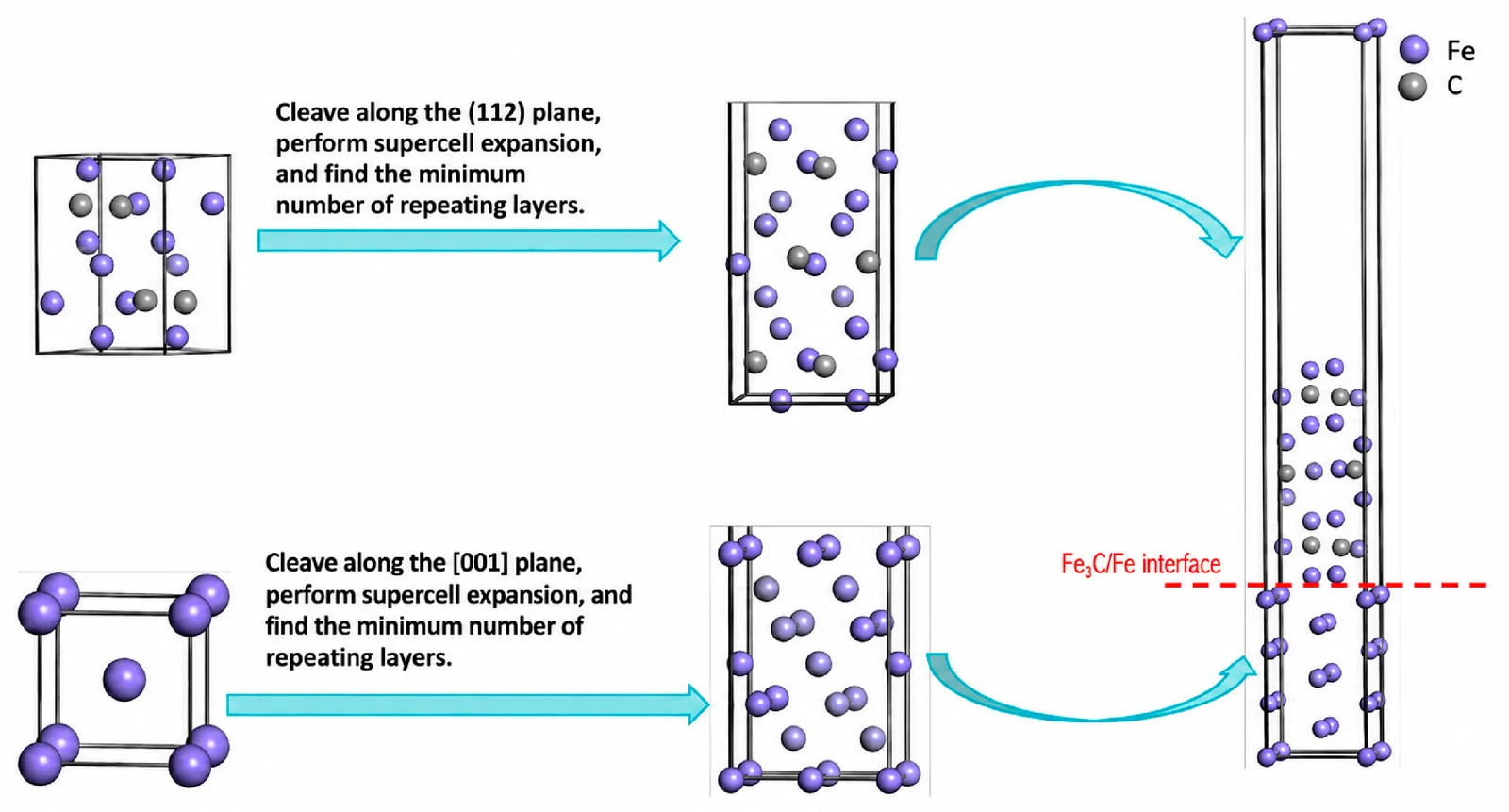
Construction process of the Fe and Fe_3_C interface model in the Bagaryatsky orientation.

**Figure 6:**
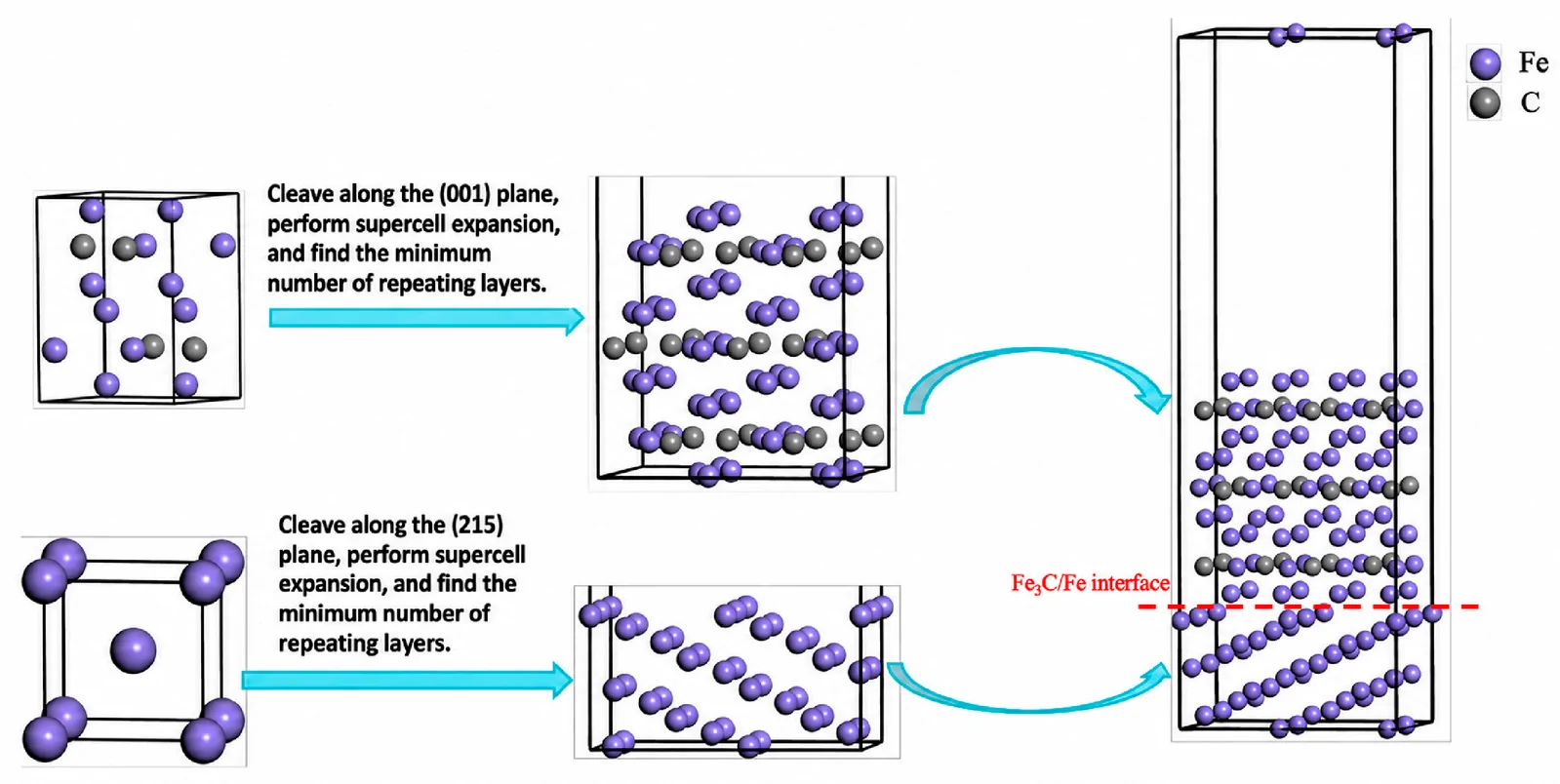
Construction process of the Fe and Fe_3_C interface model in the Pitsch-Petch orientation.

**Figure 7:**
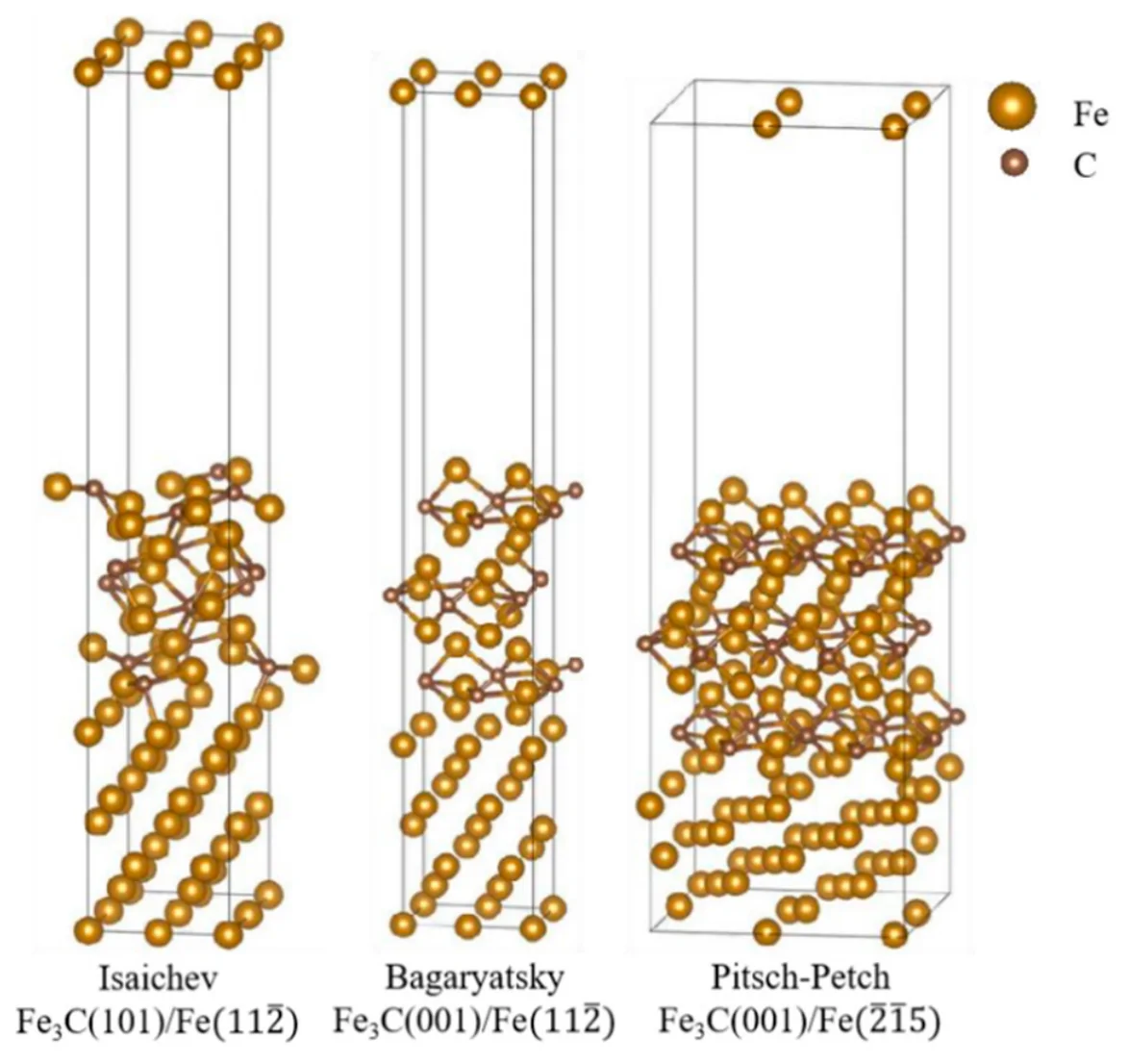
Interface models for the three different orientations.

**Figure 8:**
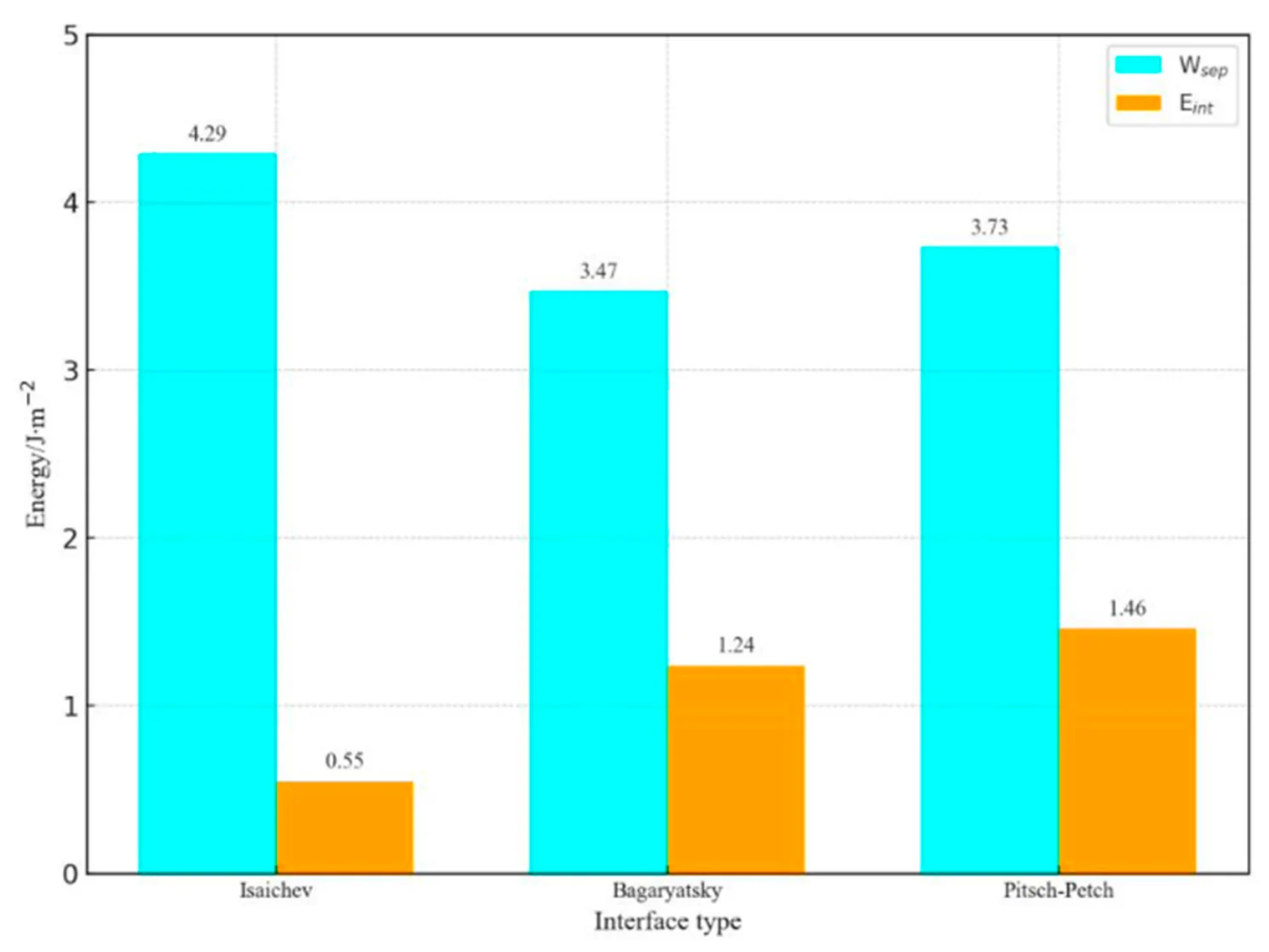
Comparison chart of interface energy (E_int_) and work of separation (W_sep_) for the three different orientation interfaces.

**Table 1:** Unit Cell Structure Parameters of α-Fe and Fe_3_C Before Structural Optimization.

Material	Crystal System	Space Group Information	Lattice Parameters
α-Fe	Body-centered Cubic	Im$\bar{3}$m	a = b = c = 2.83135 Å, α = β = γ = 90°
Fe_3_C	Orthorhombic	Pnma	a = 5.092 Å, b = 6.741 Å, c = 4.527 Å, α = β = γ = 90°

**Table 2:** Unit Cell Structure Parameters of α-Fe and Fe_3_C After Structural Optimization.

Material	Crystal System	Space Group Information	Lattice Parameters
α-Fe	Body-centered Cubic	Im$\bar{3}$m	a = b = c = 2.83135 Å, α = β = γ = 90°
Fe_3_C	Orthorhombic	Pnma	a = 5.092 Å, b = 6.741 Å, c = 4.527 Å, α = β = γ = 90°

**Table 3:** Calculated Elastic Constants of Fe_3_C (GPa).

C_11_	C_12_	C_13_	C_22_	C_23_	C_33_	C_44_	C_55_	C_66_
377.5	156.3	153.3	342.3	170.6	305.2	14.8	134.5	132.3

**Table 4:** Other Fundamental Mechanical Properties of Fe_3_C.

	Voigt	Reuss	Hill	Min	Max	Anisotropy
Bulk Modulus (GPa)	243.8	133.2	190.2	54.9	308.4	5.6
Shear Modulus (GPa)	92.6	47.6	70.1	14.8	134.5	9.1
Poisson’s Ratio	0.32	0.399	0.356	\	\	\

**Table 5:** Lattice parameters and mismatch degrees for different interfaces.

Orientation	Interface Type	Lattice Parameter/Å	ε/%
Isaichev	Fe_3_C (101)/Fe (11$\bar{2}$)	a = 8.008, b = 4.904, c = 29.98	1.712
Bagaryatsky	Fe_3_C (001)/Fe (11$\bar{2}$)	a = 4.477, b = 5.031, c = 32.112	7.212
Pitsch-Petch	Fe_3_C (001)/Fe ($\bar{2}\bar{1}$5)	a = b = 9.930, c = 29.839	12.6

**Table 6:** Interface energy and work of separation for different orientation interfaces.

Interface	E_total_ (eV)	$E_{Fe}^{slab}$ (eV)	$E_{Fe3C}^{slab}$ (eV)	A (Å^2^)	E_interface_ (J/m^2^)	W_sep_ (J/m^2^)
Isaichev	−487.36	−218.19	−258.65	39.27	0.55	4.29
Bagaryatsky	−309.38	−109.011	−195.49	22.52	1.24	3.47
Pitsch-Petch	−1102.61	−300.74	−781.36	88.09	1.46	3.72

## Data Availability

The original contributions presented in this study are included in the article. Further inquiries can be directed to the corresponding authors.

## References

[1] Wang X., Zhao J., Wang D. (2025). Iron-catalyzed sequential hydrosilylation. Nat. Commun..

[2] Jiang S., Wang H., Wu Y., Liu X., Chen H., Yao M., Gault B., Ponge D., Raabe D., Hirata A. (2017). Ultrastrong steel via minimal lattice misfit and high-density nanoprecipitation. Nature.

[3] Hou Y., Zheng Y., Ling C., Li D. (2023). Simulation study on the evolution behavior of micro pores in martensitic heat-resistant steel under multiaxial loading. J. Solid Mech..

[4] Sun B., Lu W., Gault B. (2021). Chemical heterogeneity enhances hydrogen resistance in high-strength steels. Nat. Mater..

[5] Borchers C., Kirchheim R. (2016). Cold-drawn pearlitic steel wires. Prog. Mater. Sci..

[6] Bhadeshia H. (2020). Cementite. Int. Mater. Rev..

[7] Varin R., Haftek J. (1984). Structural changes in a ferritic heat-resistant steel after long-term service. Mater. Sci. Eng..

[8] Ling S., Kaplan D.L., Buehler M.J. (2018). Nanofibrils in nature and materials engineering. Nat. Rev. Mater..

[9] Lv Z.Q., Zhang F.C., Sun S.H., Wang Z.H., Jiang P., Zhang W.H., Fu W.T. (2008). First-principles study on the mechanical, electronic and magnetic properties of Fe_3_C. Comput. Mater. Sci..

[10] Di Liberto G., Morales-García Á., Bromley S.T. (2022). An unconstrained approach to systematic structural and energetic screening of materials interfaces. Nat. Commun..

[11] Chen F., Jiang H., Zhang Y. (2023). First-principles calculation of bonding and hydrogen trapping mechanism of Fe_3_C/α-Fe interface. J. Mater. Res. Technol..

[12] Zhou Y.T., Zheng S.J., Jiang Y.X., Zhao T.Z., Wang Y.J., Ma X.L. (2017). Atomic structure of the Fe/Fe_3_C interface with the Isaichev orientation in pearlite. Philos. Mag..

[13] Guziewski M., Coleman S.P., Weinberger C.R. (2016). Atomistic investigation into the atomic structure and energetics of the ferrite-cementite interface: The Bagaryatskii orientation. Acta Mater..

[14] Ono T., Fujimoto Y., Tsukamoto S. (2012). First-principles calculation methods for obtaining scattering waves to investigate transport properties of nanostructures. Quantum Matter.

[15] Kong G.X., Ma X.J., Liu Q.J., Li Y., Liu Z.-T. (2018). Structural stability, elastic and thermodynamic properties of Au–Cu alloys from first-principles calculations. Phys. B Condens. Matter.

[16] Tannor D.J., Marten B., Murphy R., Friesner R.A., Sitkoff D., Nicholls A., Honig B., Ringnald M., Goddard W.A. (1994). Accurate first principles calculation of molecular charge distributions and solvation energies from ab initio quantum mechanics and continuum dielectric theory. J. Am. Chem. Soc..

[17] Yang N., Yang D., Zhang G. (2018). The effects of graphene stacking on the performance of methane sensor: A first-principles study on the adsorption, band gap and doping of graphene. Sensors.

[18] Meng Y.S., Arroyo-de Dompablo M.E. (2009). First principles computational materials design for energy storage materials in lithium ion batteries. Energy Environ. Sci..

[19] Ruan H., Chen Y. (2001). Ring solitons, dromons, breathers, and instantons of the (2+1)-dimensional nonlinear Schr ö dinger equation. Acta Phys. Sin..

[20] Orio M., Pantazis D.A., Neese F. (2009). Density functional theory. Photosynth. Res..

[21] Parr R.G., Yang W. (1984). Density functional approach to the frontier-electron theory of chemical reactivity. J. Am. Chem. Soc..

[22] Dal Corso A., Pasquarello A., Baldereschi A. (1996). Generalized-gradient approximations to density-functional theory: A comparative study for atoms and solids. Phys. Rev. B.

[23] Hohenberg P., Kohn W. (1964). Inhomogeneous electron gas. Phys. Rev..

[24] Rajagopal A.K., Callaway J. (1973). Inhomogeneous electron gas. Phys. Rev. B.

[25] Nityananda R. (2017). Inhomogeneous electron gas. Reson. J. Sci. Educ..

[26] Pan X.Y., Sahni V. (2003). Corollary to the Hohenberg–Kohn theorem. Int. J. Quantum Chem..

[27] Kohn W., Sham L.J. (1965). Self-consistent equations including exchange and correlation effects. Phys. Rev..

[28] Khon W., Sham L.J. (1965). Quantum density oscillations in an inhomogeneous electron gas. Phys. Rev..

[29] Liu E., Zhu B., Luo J. (2008). Semiconductor Physics.

[30] Shan B. (2015). Nanoscale Computational Simulation in Materials Science: From Basic Principles to Algorithm Implementation.

[31] Kohn W. (1999). Nobel Lecture: Electronic structure of matter-wave functions and density functionals. Rev. Mod. Phys..

[32] Wang C.S., Klein B.M., Krakauer H. (1985). Theory of magnetic and structural ordering in iron. Phys. Rev. Lett..

[33] Perdew J.P., Chevary J.A., Vosko S.H., Jackson K.A., Pederson M.R., Singh D.J., Fiolhais C. (1992). Atoms, molecules, solids, and surfaces: Applications of the generalized gradient approximation for exchange and correlation. Phys. Rev. B.

[34] Perdew J.P., Burke K., Ernzerhof M. (1996). Generalized gradient approximation made simple. Phys. Rev. Lett..

[35] Perdew J.P., Burke K., Wang Y. (1996). Generalized gradient approximation for the exchange-correlation hole of a many-electron system. Phys. Rev. B.

[36] Hammer B., Hansen L.B., Nørskov J.K. (1999). Improved adsorption energetics within density-functional theory using revised Perdew-Burke-Ernzerhof functionals. Phys. Rev. B.

[37] Titirici M.M., White R.J., Brun N., Budarin V.L., Su D.S., del Monte F., Clarkd J.H., MacLachlang M.J. (2015). Sustainable carbon materials. Chem. Soc. Rev..

[38] Guan M., Lian C., Meng S. (2016). Real time density functional theory based on atomic orbital basis: Methods and applications. Acta Phys. Sin..

[39] Phillips J.C., Kleinman L. (1959). New method for calculating wave functions in crystals and molecules. Phys. Rev..

[40] Vanderbilt D. (1990). Soft self-consistent pseudopotentials in a generalized eigenvalue formalism. Phys. Rev. B.

[41] Cohen M.L., Heine V. (1970). The fitting of pseudopotentials to experimental data and their subsequent application. Solid State Phys..

[42] Yin M.T., Cohen M.L. (1982). Theory of ab initio pseudopotential calculations. Phys. Rev. B.

[43] Bachelet G.B., Hamann D.R., Schlüter M. (1982). Pseudopotentials that work: From H to Pu. Phys. Rev. B.

[44] Hamann D.R. (1989). Generalized norm-conserving pseudopotentials. Phys. Rev. B.

[45] Shirley E.L., Allan D.C., Martin R.M. (1989). Extended norm-conserving pseudopotentials. Phys. Rev. B.

[46] Appelbaum J.A., Hamann D.R. (1973). Self-consistent pseudopotential for Si. Phys. Rev. B.

[47] Louie S.G., Schlüter M., Chelikowsky J.R., Cohen M.L. (1976). Self-consistent electronic states for reconstructed Si vacancy models. Phys. Rev. B.

[48] Marzari N., Mostofi A.A., Yates J.R., Souza I., Vanderbilt D. (2012). Maximally localized Wannier functions: Theory and applications. Rev. Mod. Phys..

[49] Laasonen K., Pasquarello A., Car R., Lee C., Vanderbilt D. (1993). Car-Parrinello molecular dynamics with Vanderbilt ultrasoft pseudopotentials. Phys. Rev. B.

[50] Kresse G., Hafner J. (1994). Norm-conserving and ultrasoft pseudopotentials for first-row and transition elements. J. Phys. Condens. Matter.

[51] Tsutsumi Y. (1987). Schrodinger equation. Funkc. Ekvacioj.

[52] Berezin F.A., Shubin M. (2012). The Schrödinger Equation.

[53] Cramer C.J. (2013). Essentials of Computational Chemistry: Theories and Models.

[54] Freysoldt C., Grabowski B., Hickel T., Neugebauer J., Kresse G., Janotti A., Van de Walle C.G. (2014). First-principles calculations for point defects in solids. Rev. Mod. Phys..

[55] Lykos P., Pratt G.W. (1963). Discussion on the Hartree-Fock approximation. Rev. Mod. Phys..

[56] Löwdin P.O. (1955). Quantum theory of many-particle systems. I. Physical interpretations by means of density matrices, natural spin-orbitals, and convergence problems in the method of configurational interaction. Phys. Rev..

[57] Akkermans R.L.C., Spenley N.A., Robertson S.H. (2013). Monte Carlo methods in materials studio. Mol. Simul..

[58] Yoon K., Rahnamoun A., Swett J.L., Iberi V., Cullen D.A., Vlassiouk I.V., Belianinov A., Jesse S., Sang X., Ovchinnikova O.S. (2016). Atomistic-scale simulations of defect formation in graphene under noble gas ion irradiation. ACS Nano.

[59] Wang V., Xu N., Liu J.C., Tang G., Geng W.-T. (2021). VASPKIT: A user-friendly interface facilitating high-throughput computing and analysis using VASP code. Comput. Phys. Commun..

[60] Timoshenko S.P., Gere J.M. (2012). Theory of Elastic Stability.

[61] Whiting E.E. (1968). An empirical approximation to the Voigt profile. J. Quant. Spectrosc. Radiat. Transf..

[62] Reuss A., Angnew Z. (1929). A calculation of the bulk modulus of polycrystalline materials. Math. Method.

[63] Hill R. (1952). The elastic behaviour of a crystalline aggregate. Proc. Phys. Soc. Sect. A.

[64] Kim J., Ghaffarian H., Kang K. (2021). The lattice dislocation trapping mechanism at the ferrite/cementite interface in the Isaichev orientation relationship. Sci. Rep..

[65] Guziewski M., Coleman S.P., Weinberger C.R. (2018). Interface energetics and structure of the pearlitic microstructure in steels: An atomistic and continuum investigation. Acta Mater..

